# Efficient Decrease in Corrosion of Steel in 0.1 M HCl Medium Realized by a Coating with Thin Layers of MnTa_2_O_6_ and Porphyrins Using Suitable Laser-Type Approaches

**DOI:** 10.3390/nano12071118

**Published:** 2022-03-28

**Authors:** Mihaela Birdeanu, Ion Fratilescu, Camelia Epuran, Alin Constantin Murariu, Gabriel Socol, Eugenia Fagadar-Cosma

**Affiliations:** 1 National Institute for Research and Development in Electrochemistry and Condensed Matter, Plautius Andronescu Street 1, 300224 Timisoara, Romania; mihaelabirdeanu@gmail.com; 2 Institute of Chemistry “Coriolan Dragulescu”, Mihai Viteazu Ave. 24, 300223 Timisoara, Romania; ionfratilescu@acad-icht.tm.edu.ro (I.F.); ecamelia@acad-icht.tm.edu.ro (C.E.); 3 National Research & Development Institute for Welding and Material Testing—ISIM, Mihai Viteazu Ave. 30, 300222 Timisoara, Romania; amurariu@isim.ro; 4 National Institute for Laser, Plasma and Radiation Physics, 409 Atomiştilor Street, 077125 Măgurele, Romania; gabriel.soco@inflpr.ro

**Keywords:** MnTa_2_O_6_, porphyrins, sandwich thin layers, PLD and MAPLE laser deposition, AFM and SEM microscopy, corrosion measurements, coating performance

## Abstract

The purpose of this research is to meet current technical and ecological challenges by developing novel steel coating systems specifically designed for mechanical equipment used in aggressive acid conditions. Homogeneous sandwich-type layered films on the surface of steel electrodes were realized using a pseudo-binary oxide, MnTa_2_O_6_, and two different substituted porphyrin derivatives, namely: 5-(4-carboxy-phenyl)-10,15,20-tris (4-methyl-phenyl)-porphyrin and 5-(4-methyl-benzoate)-10,15,20-tris (4-methyl-phenyl)-porphyrin, which are novel investigated compound pairs. Two suitable laser strategies, pulsed laser deposition (PLD) and matrix-assisted pulsed laser evaporation (MAPLE), were applied in order to prevent porphyrin decomposition and to create smooth layers with low porosity that are extremely adherent to the surface of steel. The electrochemical measurements of corrosion-resistant coating performance revealed that in all cases in which the steel electrodes were protected, a significant value of corrosion inhibition efficiency was found, ranging from 65.6 to 83.7%, depending on the nature of the porphyrin and its position in the sandwich layer. The highest value (83.7%) was obtained for the MAPLE/PLD laser deposition of 5-(4-carboxy-phenyl)-10,15,20-tris (4-methyl-phenyl)-porphyrin/MnTa_2_O_6_(h), meaning that the inhibitors adsorbed and blocked the access of the acid to the active sites of the steel electrodes.

## 1. Introduction

At present, due to its adjustable mechanical properties, steel represents the most reliable metallic material in several industrial fields, such as: petrochemical, infrastructure, construction and building, renewable energy, automotive and aerospace sectors. Because of the need to function in different harsh environments, steel equipment requires protection against corrosion in order to fulfill requirements for safety and long life cycles [1,2].

Developing coatings with improved properties to achieve resilience, increased service life of mechanical equipment and minimal environmental impact is a current demand worldwide. Current effective strategies used to protect steel substrates include advanced electroplating methods such as long pulse plating to realize Al and Zn alloy electrodeposited coatings free of toxic Cd, Co and Cr compounds [3,4] or to cover surfaces with organic and inorganic corrosion inhibitors [5,6,7,8].

Laser-type technologies are recognized as versatile methods for the deposition of different types of organic and inorganic coatings on steel [9], thus increasing their resistance against corrosion and high-temperature oxidation.

Well known for their optoelectronic properties, porphyrins are used in sensor formulations [10], for proton exchange membrane fuel cells [11] and as catalysts [12], but they also proved to act as highly effective corrosion inhibitors [13,14,15,16,17,18].

Usually, these heterocyclic compounds operate on the metal surface by adsorption, thus blocking the active sites responsible for corrosion processes [16]. The nature, position and volume of different substituents on the porphyrin molecule can determine the generation of a more or less compact layer [19]. For instance, para-amino substituents adopt a flat structure on the surface, but ortho-amino substituents adopt a saddle shape orientation and are thus capable of being stacked closely to each other [20].

Among the most used inorganic compounds to protect steel from corrosion are oxides [21] and pseudo-binary oxides [22,23,24].

In the last decade, our group has conducted successful research regarding the synergism between pseudo-binary oxides and porphyrins or metalloporphyrins deposited in alternating sandwich layers on steel with the purpose of inhibiting its corrosion even in extremely aggressive acidic environments. The corrosion inhibition mechanism was achieved by covering the steel surfaces with extremely adherent mixed layers of pseudo-binary oxides and porphyrins, thus creating a protective mechanical barrier [25,26,27,28].

**Figure 1:**
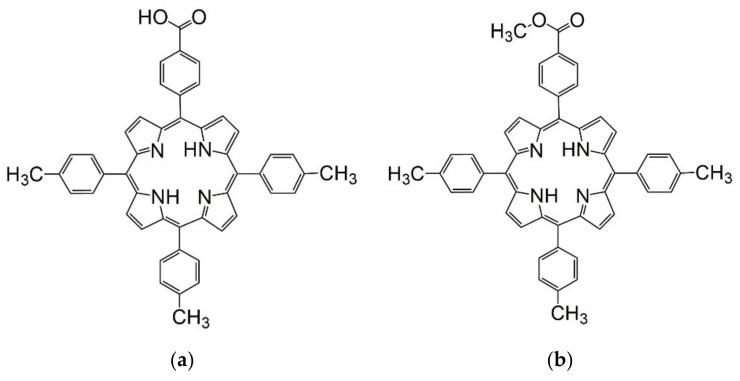
Chemical structures of 5-(4-carboxy-phenyl)-10,15,20-tris (4-methyl-phenyl)-porphyrin (**a**) and 5-(4-methyl-benzoate)-10,15,20-tris (4-methyl-phenyl)-porphyrin (**b**).

The purpose of this research is to meet technical, ecological and sustainability challenges by systematically developing novel steel coating systems specifically for mechanical equipment used in aggressive acid conditions. Uniform, durable and homogeneous composite well-packed films on the surface of steel electrodes [29] were achieved by specific laser strategies using a pseudo-binary oxide, MnTa_2_O_6_, and two different substituted porphyrins derived from one another, namely: 5-(4-carboxy-phenyl)-10,15,20-tris (4-methyl-phenyl)-porphyrin and 5-(4-methyl-benzoate)-10,15,20-tris (4-methyl-phenyl)-porphyrin, which are novel investigated compound pairs (structures in Figure 1a,b).

The porphyrins were selected starting from the knowledge that in the case of other corrosion inhibitors, the presence of carboxyl, hydroxyl or ester functional groups [30] favor electrostatic interactions with the metal surface. The characterization and evaluation of the developed coatings in acid media show that these inorganic/organic coverings can protect the steel surface from the surrounding reactive environment.

## 2. Materials and Methods

Corrosion inhibition was achieved by covering steel surfaces with thin films of both porphyrin and MnTa_2_O_6_ in different deposition orders. Two suitable laser strategies were used in order to prevent porphyrin decomposition and to create smooth layers with low porosity that are extremely adherent to the surface of steel.

### 2.1. Obtaining Pseudo-Binary Oxides and Porphyrins

The nanomaterials that were investigated in order to confirm their corrosion inhibition capacity are classified as pseudo-binary oxides and porphyrins.

#### 2.1.1. Obtaining and Characterizing MnTa_2_O_6_

As an inorganic component, the mixed oxide MnTa_2_O_6_ was obtained by the hydrothermal synthesis method (MnTa_2_O_6_(h)).

The precursors used for hydrothermal synthesis of MnTa_2_O_6_(h) were: manganese (II) oxide (MnO) (99.99%, Merck) and tantalum (V) oxide (Ta_2_O_5_) (99.99%, Merck Millipore, Darmstadt, Germany) in the molar ratio 1:1, while the pH was stabilized at 12 by the addition of powdered NaOH (99%, Sigma-Aldrich, St. Louis, MO, USA). The mixture was transferred to a Teflon-lined stainless steel 316 L autoclave (70 mL capacity) that was charged to 80% of the autoclave total volume capacity for 12 h at 240 °C. The precipitate obtained from the hydrothermal synthesis was washed on filter paper with double-distilled water and ethyl alcohol (99.6%, Merck Millipore, Darmstadt, Germany).

**Figure 2:**
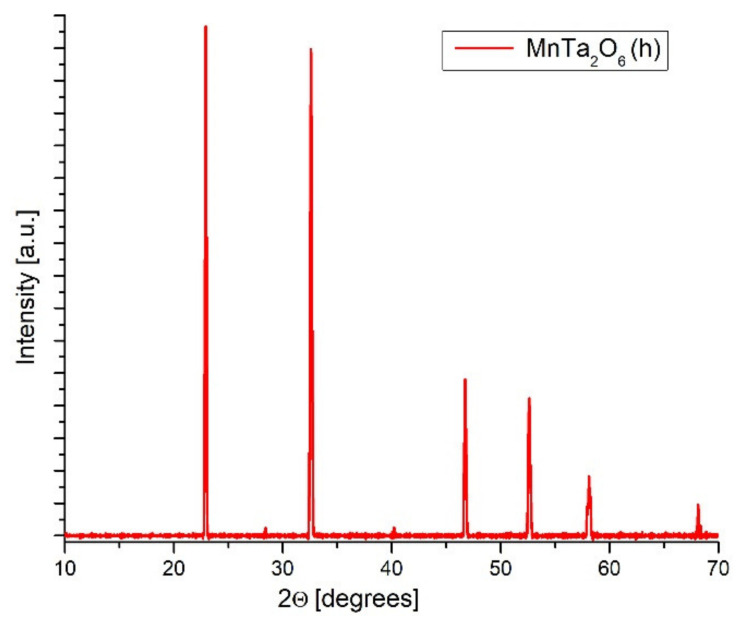
XRD patterns of MnTa_2_O_6_(h).

Figure 2 presents the X-ray diffraction patterns for MnTa_2_O_6_(h). The peaks of MnTa_2_O_6_(h) nanomaterials were identified using JCPDS, card no. 01-072-0485, belonging to the *Pbcn* space group. The highest intensity peak is located at 2θ = 23.8°, and it is attributed to the (311) plane. The intensity of the peaks from the X-ray diffraction spectrum demonstrates the very good quality and high purity of the MnTa_2_O_6_(h) nanomaterial obtained by the hydrothermal synthesis method.

Using the computer package FullProf Suite, the lattice constants were calculated from XRD analysis data for MnTa_2_O_6_(h): elementary cell volume V/10^6^/pm^3^ = 429.14 and Miller indices a = 14.42 Å, b = 5.79 Å, c = 5.14 Å, and α = β = γ = 90°.

**Figure 3:**
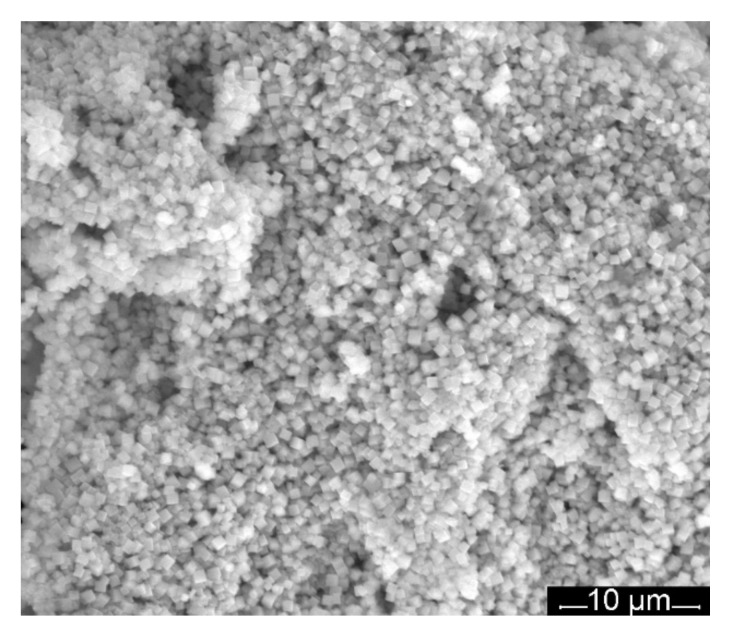
SEM micrographs of MnTa_2_O_6_(h).

Figure 3 shows the SEM morphology of the MnTa_2_O_6_(h) oxides obtained by the hydrothermal method. The image shows that it consists of small identical cube crystals.

#### 2.1.2. Obtaining Porphyrins

The porphyrins were obtained by two-step organic synthesis. The first step consists of a multicomponent condensation reaction [31] between a mixture of two aldehydes: methyl 4-formylbenzoate (99%, Sigma-Aldrich, St. Louis, MO, USA) and *p*-tolualdehyde (97%, Sigma-Aldrich, St. Louis, MO, USA) in a molar ratio of 1:3 and the corresponding amount of pyrrole (98%, Sigma-Aldrich, St. Louis, MO, USA), all solved in propionic acid (99.5%, Sigma-Aldrich, St. Louis, MO, USA), destined to form the methyl ester of 5-(4-carboxy-phenyl)-10,15,20-tris (4-methyl-phenyl)-porphyrin. This step was followed by the second hydrolysis step with potassium hydroxide (KOH) (min. 85%, Merck Millipore, Darmstadt, Germany) in ethylic alcohol (EtOH) (97%, Sigma-Aldrich, St. Louis, MO, USA) to generate the COOH-porphyrin derivative [32].

### 2.2. Data on the Selected Steel Substrate

The substrate material selected for these experiments is martensitic stainless-steel bar 1.4034/AISI 420/X46Cr13 with a diameter of 10 mm. Martensitic stainless steels have sufficient corrosion resistance in water or steam, alkaline media, and fruit and vegetable juices, but their corrosion resistance is limited in a chloride or acidic environment, in contrast to austenitic or precipitation hardening stainless steels due to their high carbon content [33].

Martensitic stainless steels are cheap, have very high hardness and strong mechanical properties and thus are ideal for equipment working in conditions where wear resistance is required (surgical instruments, cutting tools, brake discs, pump components, turbines, roller bearings, press plates, skates, etc.).

The corrosion resistance of unprotected martensitic stainless steels varies significantly depending on chemical composition and the quality of surface finishing. They can be highly polished to a mirror finish. Austenitizing stainless steel results in different microstructural changes that might weaken corrosion resistance [34]. On the contrary, the dissolution of chrome carbides that generate higher amounts of dissolved chrome and carbon might lead to a passive layer richer in chrome and, as a consequence, provide increased corrosion resistance. In its delivered state, 1.4034 martensitic steel is not resistant to intergranular corrosion [35].

### 2.3. Apparatus

The purity of the synthesized MnTa_2_O_6_(h) powder was identified using X-ray diffraction (XRD) on a PW 3040/60 X’Pert PRO Powder Diffractometer with monochromatic incident radiation Cu Kα (λ = 1.5418 Ǻ).

Field-emission scanning electron microscopy (SEM)/EDAX (model IN-SPECT S) and atomic force microscopy (AFM) apparatus (Nanosurf^®^EasyScan2, Nanosurf AG, Liestal, Switzerland) provided data for morphologic characterization of the thin films. SEM analyses were performed with a magnification = 6000 ×, weight distance = 10.4 mm and high voltage = 30.00 kV at low vacuum.

Using the Nanosurf^®^EasyScan 2 software for samples scanned in noncontact mode (scan size of 1.15 μm × 1.15 μm; points/line = 1024; time/line = 1 s) and the equations reported in [36], nano-roughness was calculated for each sample.

For the deposition of the thin layers, two procedures were chosen: pulsed laser deposition (PLD) and matrix-assisted pulsed laser evaporation (MAPLE). The MAPLE process is indicated if biomaterials or degradable polymers are used that may undergo structural changes at the temperatures imposed by the pulsed laser deposition (PLD) technique and was selected for each porphyrin covering.

The monolayer and sandwich thin-film depositions (PLD and MAPLE) were performed by Lambda Physics Coherent—exciter laser source, COMPexPro 205 F model.

The parameters used for the MAPLE technique were: pressure in the vacuum chamber = 10^−3^ mbar; distance between target and substrates (d) = 50 mm; pulse energy (E) = 410 mJ (75 mJ in chamber); temperature (T) = room temperature; number of pulses (N) = 80,000 (the frequency 20 Hz).

The parameters used in the case of the PLD technique (selected for inorganic oxide MnTa_2_O_6_(h)) were: pressure in the vacuum chamber in an atmosphere with O_2_ = 1.5 × 10^−1^ mbar; distance between target and substrates (d) = 50 mm; pulse energy (E) = 400 mJ (280 mJ in chamber); temperature (T) = room temperature; number of pulses (N) = 2000 (the frequency 10 Hz).

For the electrochemical tests, the Voltalab (model PGZ 402, Radiometer Analytical – Copenhagen, Denmark) potentiostat was used, combined with VoltaMaster 4 software v.7.09 for interpreting the results. The corrosion inhibition properties of the deposited thin films were evaluated through electrochemical measurements. The setup of the electrochemical measurements included an electrochemical cell with three electrodes, i.e., the evaluated specimen (either a bare electrode (OL) for the control or modified by covering it with laser steel disks), a platinum wire counter electrode and a saturated calomel electrode (SCE) as the standard reference electrode, connected to the previously described potentiostat. The standard hydrogen electrode (SHE) was the reference for all of the potentials that are discussed in the current paper. The potential range scanned during the potentiodynamic polarization measurements was from −1.3 V to −0.6 V using a 1 mV/s scan rate while maintaining the temperature at 23 °C ± 1 °C. Before the polarization, the open-circuit potential (OCP) was monitored for 30 min. A 0.28 cm^2^ constant active surface of the steel samples was ensured by mounting the disks in a Teflon body before submerging the electrode into the HCl solution. For calculating the values of corrosion potential (Ecorr), corrosion current density (icorr), polarization resistance (Rp), corrosion rate (νcorr), the anodic Tafel slope (βa) and the cathodic Tafel slope (βc), i.e., the Tafel parameters, VoltaMaster 4 v. 7.09 software was used. The equation in [37] was applied for determining the corrosion inhibition efficiency (IE%).

### 2.4. Design of Steel Coverings

Thin films of 5-(4-carboxy-phenyl)-10,15,20-tris (4-methyl-phenyl)-porphyrin and 5-(4-methyl-benzoate)-10,15,20-tris (4-methyl-phenyl)-porphyrin were obtained using the non-destructive MAPLE technique, while the thin films with pseudo-binary oxides (MnTa_2_O_6_(h)) were obtained by the PLD technique.

The thin films were deposited on 1.4034 martensitic steel with a chemical composition (wt.%) of C = 0.43–0.5; Si = 1; Mn = 1; P = 0.04; S = 0.015; Cr = 12.5 − 14.5; and Fe = 83−85, according to inspection certificate 3.1 no. 84078864000010/28.02.2019 issued by UGITECH S.A., France. The order of deposition of the inhibitors on W1.4043 steel is presented in Table 1.

## 3. Results and Discussion

### 3.1. Electrochemical Test

**Figure 4:**
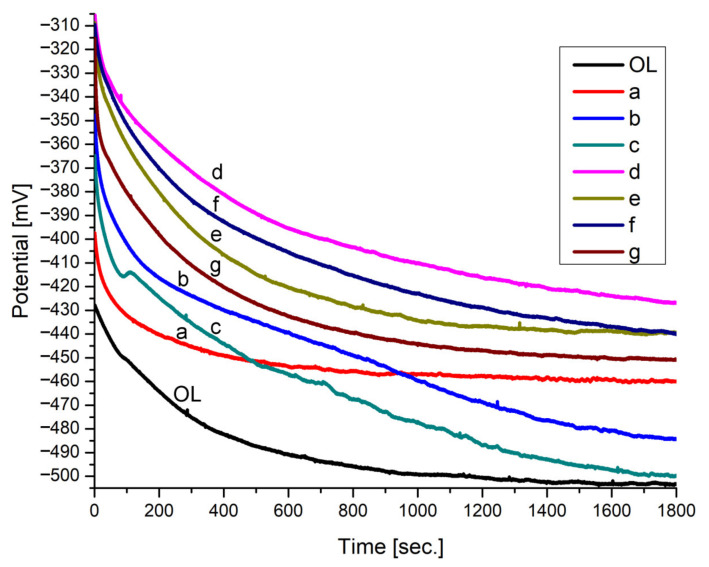
The OCP of the thin film electrodes immersed in 0.1 M HCl: OL bare W1.4043: (a) MnTa_2_O_6_(h); (b) 5-(4-carboxy-phenyl)-10,15,20-tris (4-methyl-phenyl)-porphyrin; (c) 5-(4-methyl-benzoate)-10,15,20-tris (4-methyl-phenyl)-porphyrin; (d) 5-(4-carboxy-phenyl)-10,15,20-tris (4-methyl-phenyl)–porphyrin/MnTa_2_O_6_(h); (e) MnTa_2_O_6_(h)/5-(4-carboxy-phenyl)-10,15,20-tris (4-methyl-phenyl)-porphyrin; (f) 5-(4-methyl-benzoate)-10,15,20-tris (4-methyl-phenyl)-porphyrin/MnTa_2_O_6_(h); (g) MnTa_2_O_6_(h)/5-(4-methyl-benzoate)-10,15,20-tris (4-methyl-phenyl)-porphyrin.

The electrochemical measurements were performed in an acidic medium (0.1 M HCl) for 30 min. From the registered open-circuit potential (OCP) slopes, it can be concluded that the OCP is stabilized after approximately 1800 s for all protected electrodes. The OCP measurements (Figure 4) show that the covered electrodes stabilize around 500–800 s, while the uncoated electrode stabilizes around 1800 s. The OCP is a qualitative indicator, and it determines the immersion time required to reach the steady state. The shift to more positive values of the OCP of the film-modified electrodes, compared with the control steel electrode (OL) (Figure 4), is a general characteristic. This behavior shows that the coatings enhance the passivation state of the carbon steel surface.

**Figure 5:**
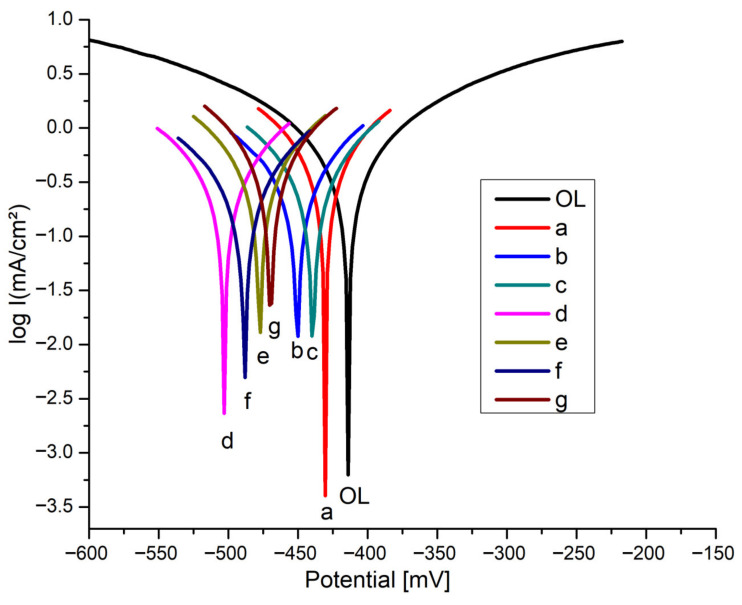
Tafel polarization curves registered in 0.1 M HCl media for the studied thin films: OL bare W1.4043; (a) MnTa_2_O_6_(h); (b) 5-(4-carboxy-phenyl)-10,15,20-tris (4-methyl-phenyl)-porphyrin; (c) 5-(4-methyl-benzoate)-10,15,20-tris (4-methyl-phenyl)-porphyrin; (d) 5-(4-carboxy-phenyl)-10,15,20-tris (4-methyl-phenyl)-porphyrin/MnTa_2_O_6_(h); (e) MnTa_2_O_6_(h)/5-(4-carboxy-phenyl)-10,15,20-tris (4-methyl-phenyl)-porphyrin; (f) 5-(4-methyl-benzoate)-10,15,20-tris (4-methyl-phenyl)-porphyrin/MnTa_2_O_6_(h); (g) MnTa_2_O_6_(h)/5-(4-methyl-benzoate)-10,15,20-tris (4-methyl-phenyl)-porphyrin.

The Tafel plots (potential vs log. current density) of the covered steel electrodes recorded in corrosive acid solution, are presented in Figure 5.

The data in Table 2 reveal that both the i_corr_ and *v*_corr_ values of the covered electrodes are smaller than those measured for the unprotected electrode. The corrosion behavior of the passivated carbon steel electrodes with porphyrin/oxide sandwich layers shows a higher corrosion resistance compared to the bare carbon steel electrode.

In all cases in which the steel electrodes were modified with thin films consisting of pseudo-binary oxides and porphyrins, a significant value of corrosion inhibition efficiency was found, ranging from 65.6 to 83.7%, depending on the nature of the porphyrin and its position in the sandwich layer. The highest value (83.7%) was obtained for the MAPLE/PLD laser deposition of 5-(4-carboxy-phenyl)-10,15,20-tris (4-methyl-phenyl)-porphyrin/MnTa_2_O_6_(h). In addition, this porphyrin also yielded the best result (73.6%) when compared with the samples protected with only one of the three inhibiting compounds.

In this case, the pseudo-binary oxide yielded the lowest result (65.6%), but its synergistic action as the upper layer on porphyrin is worth mentioning.

It seems that regardless of the structure of the porphyrin derivative (acid or ester), it is important to first cover the steel with porphyrin. In this sense, the second performance regarding corrosion inhibition was obtained by the methyl ester of 5-(4-carboxy-phenyl)-10,15,20-tris (4-methyl-phenyl)-porphyrin, also deposited on steel as the first layer.

As in the case of deposits realized by the drop-casting technique [26], with the protective layers formed by alternative MAPLE and PLD laser methods, the polarization resistance (R_p_) of the modified OL steel electrodes increased. The highest polarization resistance (R_p_) was obtained for the composite layers 5-(4-carboxy-phenyl)-10,15,20-tris (4-methyl-phenyl)-porphyrin/MnTa_2_O_6_(h), meaning that the porphyrin/oxide inhibitors adsorbed and blocked access to the active sites of the steel electrodes [38,39].

From Table 2, it can be seen that the β_c_ cathodic Tafel slopes as well as the β_a_ anodic Tafel slopes decrease significantly (more than 3 times) for all electrodes covered with thin films, regardless of whether they are a monolayer or double layers. As previously reported in [40], this decrease in the corrosion current density might be connected to the protection of steel anodic dissolution, accompanied by the similar activity of H^+^ ion cathodic reduction. Similar shifts can be observed on the cathodic and anodic branches, so we can classify the tested corrosion inhibitors into the group of mixed-type inhibitors with the ability to control both anodic and cathodic reactions.

### 3.2. Microscopic Characterization

**Figure 6:**
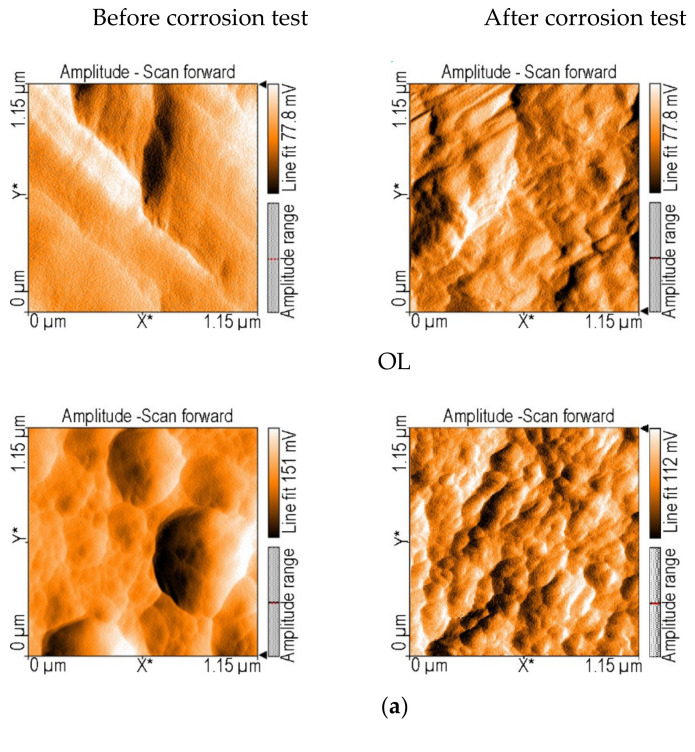

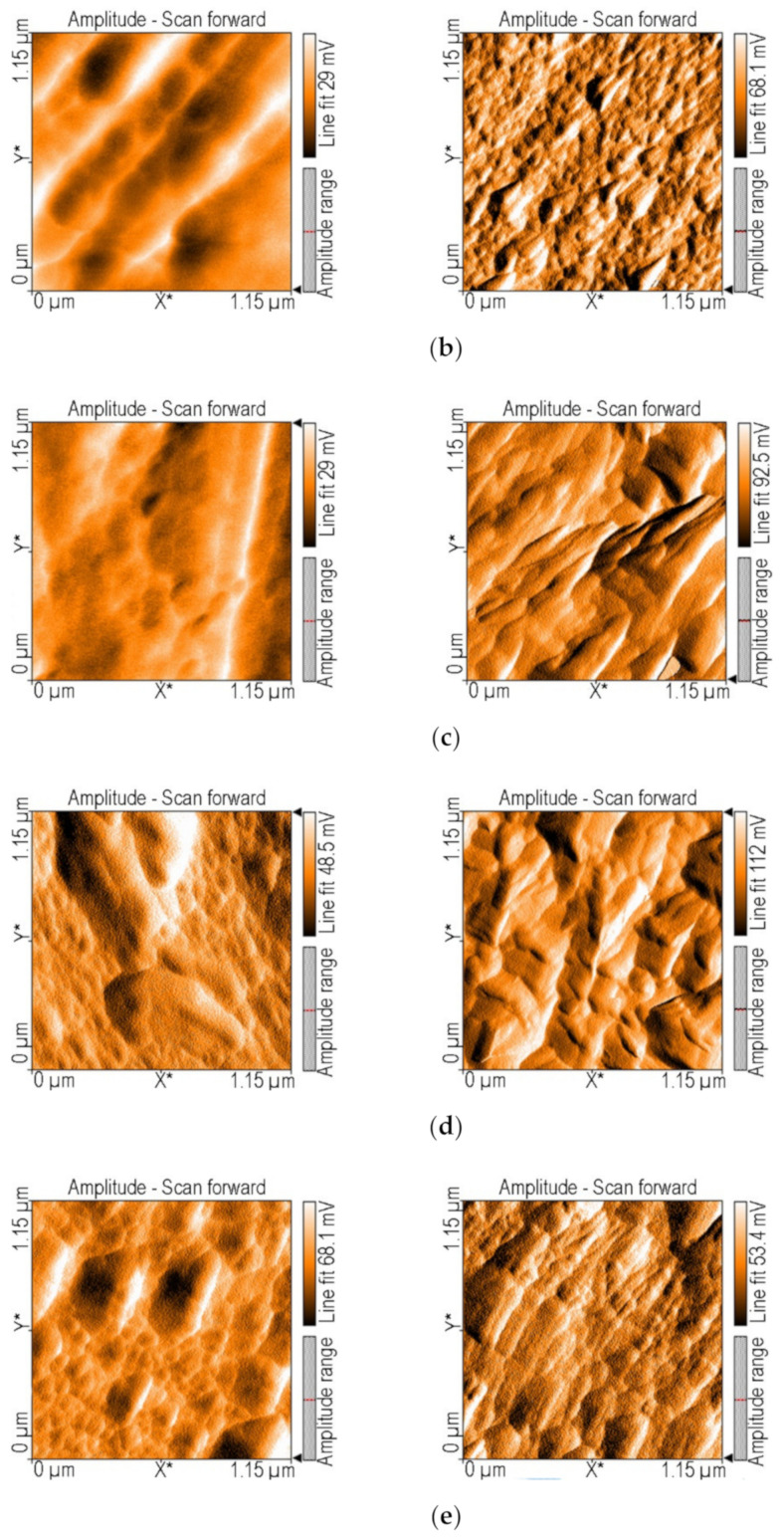

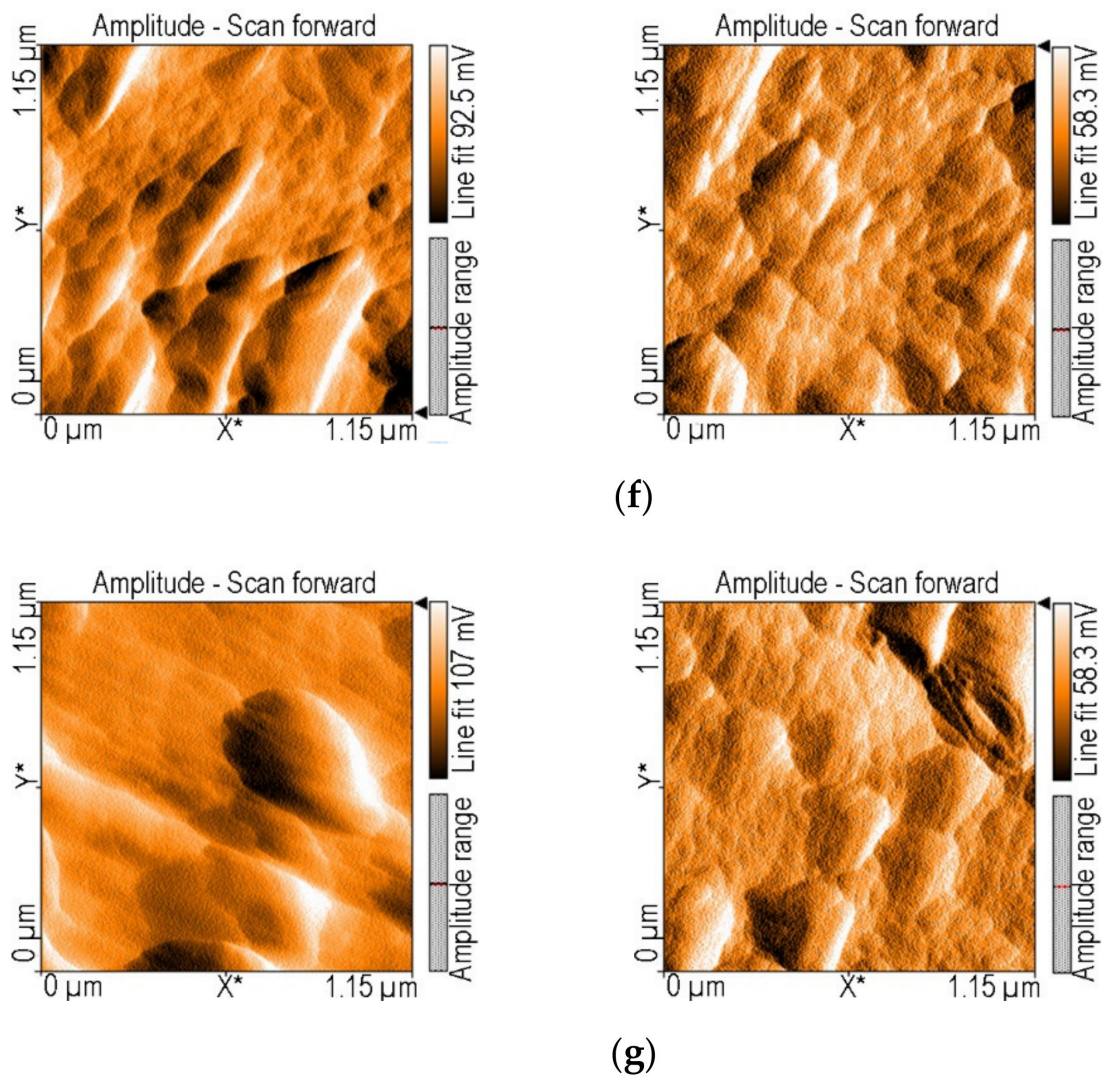
Two-dimensional AFM images for the covering layers, before and after corrosion tests: OL bare W1.4043; (**a**) MnTa_2_O_6_(h); (**b**) 5-(4-carboxy-phenyl)-10,15,20-tris (4-methyl-phenyl)-porphyrin; (**c**) 5-(4-methyl-benzoate)-10,15,20-tris (4-methyl-phenyl)-porphyrin; (**d**) 5-(4-carboxy-phenyl)-10,15,20-tris (4-methyl-phenyl)-porphyrin/MnTa2O6(h); (**e**) MnTa_2_O_6_(h)/5-(4-carboxy-phenyl)-10,15,20-tris (4-methyl-phenyl)-porphyrin; (**f**) 5-(4-methyl-benzoate)-10,15,20-tris (4-methyl-phenyl)-porphyrin/MnTa2O6(h); (**g**) MnTa_2_O_6_(h)/5-(4-methyl-benzoate)-10,15,20-tris (4-methyl-phenyl)-porphyrin.

The thin monolayer or sandwich films were analyzed from the morphological point of view using AFM microscopy, and their appearance is shown in Figure 6 (before and after corrosion tests).

From the AFM investigations (Figure 6), it can be seen that after contact with an acidic environment, the surfaces of the thin films are all restructured. The materials that came into direct contact with the corrosive environment underwent changes in their morphology, and with no exception, the size of the aggregates decreased. A common characteristic of these self-assembled structures is their triangular building blocks that generate rows, which are highly oriented in the same way and lack voids. This kind of compact and adherent covering (no pores, voids or cracks were detected) might realize physical protection by avoiding direct contact between the aggressive acid medium and the metal surface.

Using Nanosurf^®^EasyScan 2 software, S_a_ (average roughness), S_q_ (square root roughness), S_y_ (thickness of the layers) and particle size were calculated for each sample (Table 3).

From Table 3, it can be seen that the smallest particle size on the deposited surface is 35.5 nm in the case of 5-(4-carboxy-phenyl)-10,15,20-tris (4-methyl-phenyl)-porphyrin/MnTa_2_O_6_(h), which also has the smallest difference in the nano-roughness value measured before and after corrosion tests (<20 nm). This result shows that the thickness of the best protective sandwich layers is preserved during the action of the corrosive acid medium.

**Figure 7:**
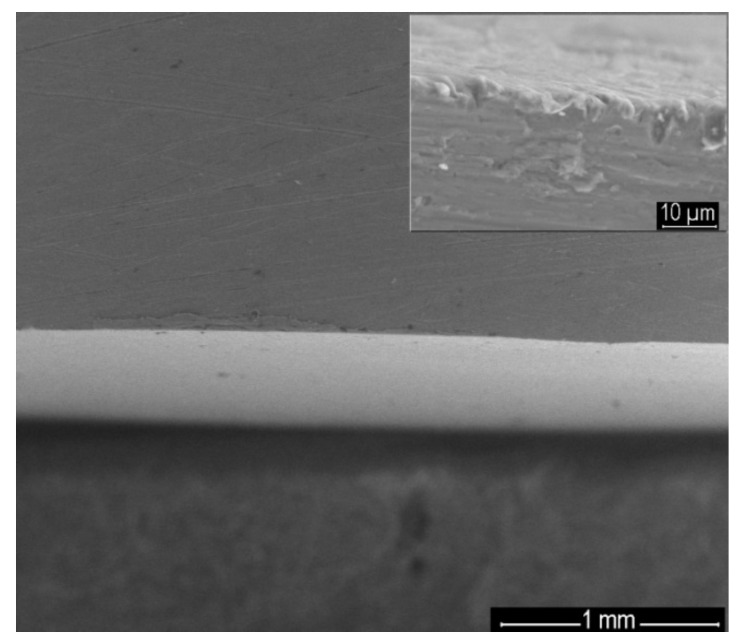
SEM image of the vertical section of the covered steel electrode with 5-(4-carboxy-phenyl)-10,15,20-tris (4-methyl-phenyl)-porphyrin/MnTa_2_O_6_(h).

The SEM image of the perpendicular section of the steel electrode covered with 5-(4-carboxy-phenyl)-10,15,20-tris (4-methyl-phenyl)-porphyrin/MnTa_2_O_6_(h) clearly shows the compact and uniform coverage of the material, with thickness ranging uniformly between 20 and 30 microns (Figure 7).

**Figure 8:**
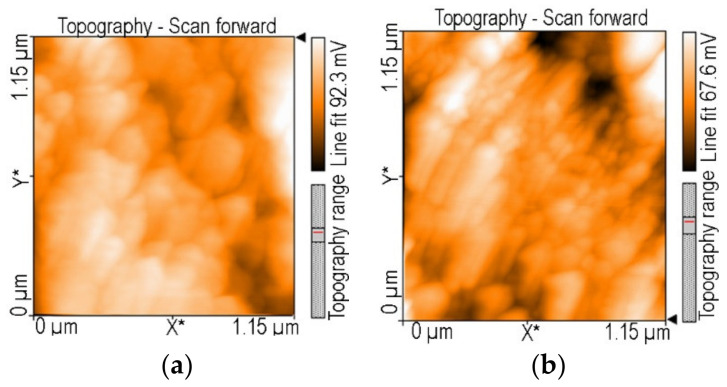
AFM topography images after corrosion tests of the steel electrodes covered with: (**a**) 5-(4-carboxy-phenyl)-10,15,20-tris (4-methyl-phenyl)-porphyrin/MnTa_2_O_6_(h) and (**b**) MnTa_2_O_6_(h), showing that pitting corrosion is absent in (**a**) and is present in (**b**).

The AFM topography for the two bordering samples, the first providing the best corrosion inhibition efficiency, namely, 5-(4-carboxy-phenyl)-10,15,20-tris (4-methyl-phenyl)-porphyrin/MnTa_2_O_6_(h) (Figure 8a), and the second, MnTa_2_O_6_(h), producing the lower value of corrosion inhibition efficiency, was investigated with the purpose of assessing their susceptibility to pitting corrosion. It is known that the combination of stainless steel with hydrochloric acid can lead to this type of attack, and we are also aware that lowering the corrosion rate is only a solution to part of the problem. The best combination, MAPLE/PLD laser deposition of 5-(4-carboxy-phenyl)-10,15,20-tris (4-methyl-phenyl)-porphyrin/MnTa_2_O_6_(h), which offered 83.7% corrosion inhibition, was not affected by pitting corrosion. No deep pores were observed (Figure 8a) in the AFM topography investigations, but the single layer of MnTa_2_O_6_, deposited by PLD, showed deep pores (up to the metal surface) in the AFM image (Figure 8b), a sign that pitting corrosion took place. As a reliable conclusion, in order to function as efficient protecting layers, sandwich layers of porphyrin/oxide compounds are necessary.

## 4. Conclusions

Novel steel coating systems based on oxides and porphyrins were specifically designed for mechanical equipment used in aggressive acid conditions. Sandwich-type structured films on the surface of the steel electrodes were obtained using a pseudo-binary oxide, MnTa_2_O_6_, and two different substituted porphyrin derivatives, namely: 5-(4-carboxy-phenyl)-10,15,20-tris (4-methyl-phenyl)-porphyrin and its methyl ester 5-(4-methyl-benzoate)-10,15,20-tris (4-methyl-phenyl)-porphyrin, which are novel investigated compound pairs. Two suitable laser strategies, pulsed laser deposition (PLD) and matrix-assisted pulsed laser evaporation (MAPLE), were used in order to prevent porphyrin decomposition and to realize homogeneous layers with low porosity that are extremely adherent to the surface of steel. The electrochemical measurements of the corrosion-resistant performance of these coatings revealed that in all cases in which the steel electrodes were protected, a significant value of corrosion inhibition efficiency was found, ranging from 65.6 to 83.7%, depending on the nature of the porphyrin and its position in the sandwich layer. The highest value (83.7%) was obtained for the MAPLE/PLD laser deposition of 5-(4-carboxy-phenyl)-10,15,20-tris (4-methyl-phenyl)-porphyrin/MnTa_2_O_6_, meaning that the inhibitors adsorbed and blocked the access of the acid to the active sites of the steel electrodes. Due to the comparable shifts observed on the cathodic and anodic branches, it can be inferred that the tested corrosion inhibitors belong to the group of mixed-type inhibitors with the ability to control both anodic and cathodic reactions. No physical stress linked to laser deposition conditions or to a mismatch between the layered structure and the steel surface could be identified, so this double combination of compounds and laser methods is a success. These materials, which are compounds with unique properties, combine synergistically into a uniform and adherent mixed layer with a thickness ranging from 20 to 30 µm. It seems that regardless of the structure of the porphyrin derivative (acid or ester), it is important to first cover the steel with porphyrin.

## Figures and Tables

**Table 1:** Nature of the inhibitors and their order of deposition on W1.4043 steel.

Sample	The Order of Covering the Steel Surface	Deposition Mode
a	MnTa_2_O_6_(h)	Monolayer/PLD
b	5-(4-carboxy-phenyl)-10,15,20-tris (4-methyl-phenyl)-porphyrin	Monolayer/MAPLE
c	5-(4-methyl-benzoate)-10,15,20-tris (4-methyl-phenyl)-porphyrin	Monolayer/MAPLE
d	5-(4-carboxy-phenyl)-10,15,20-tris (4-methyl-phenyl)-porphyrin/MnTa_2_O_6_(h)	Sandwich/ MAPLE/PLD
e	MnTa_2_O_6_(h)/5-(4-carboxy-phenyl)-10,15,20-tris (4-methyl-phenyl)-porphyrin	Sandwich /PLD/MAPLE
f	5-(4-methyl-benzoate)-10,15,20-tris (4-methyl-phenyl)-porphyrin/MnTa_2_O_6_(h)	Sandwich MAPLE/PLD
g	MnTa_2_O_6_(h)/5-(4-methyl-benzoate)-10,15,20-tris (4-methyl-phenyl)-porphyrin	Sandwich PLD/MAPLE

**Table 2:** The most significant parameters obtained from Tafel curves for protected steel electrodes after immersing them for 30 min in 0.1 M HCl medium.

Sample	E (I = 0) (mV)	R_p_ (Ωxcm^2^)	i_corr_ (mA/cm^2^)	β_a_ (mV)	β_c_ (mV)	*v*_corr_ (mm/Y)	IE (%)
OL	−414.1	88.53	1.2924	258.0	−263.5	1.511	-
a	−430.0	130.72	0.4440	84.1	−85.9	0.5192	65.64
b	−449.9	139.39	0.3401	79.2	−81.1	0.3977	73.68
c	−440.0	131.76	0.4118	82.7	−84.1	0.5067	68.13
d	**−502.8**	**159.67**	**0.2101**	**71.5**	**−72.4**	**0.2458**	**83.74**
e	−477.1	150.44	0.2573	75.8	−77.2	0.3010	80.09
f	−488.4	154.53	0.2380	73.2	−74.8	0.2784	81.58
g	−469.8	146.42	0.2677	77.3	−79.3	0.3131	79.28

**Table 3:** The dimensions of the particles and the nano-roughness before and after corrosion tests.

Sample	Area (pm^2^)	S_a_ Before/After (nm)	S_q_ Before/After (nm)	S_y_ Before/After (nm)	Particle Dimensions Before/After (nm)
OL	1.326	1.7956/51.0779	2.3321/74.8436	-	-
a		5.0354/47.8121	6.0733/70.1462	41.003/199.116	81.8/48.3
b		5.4929/43.5298	7.2987/65.9352	51.656/161.129	65.8/39.9
c		5.2775/46.0276	6.9578/68.6247	41.598/182.468	76.1/42.6
d		10.978/30.5807	16.134/49.5292	99.116/103.435	35.5/18.2
e		7.3613/38.6299	9.5030/52.7320	81.798/135.338	47.8/30.5
f		8.8238/35.3781	13.024/50.0736	95.753/113.598	43.6/24.8
g		6.6588/37.0684	8.6253/59.6374	59.809/153.524	57.4/35.7

## Data Availability

Each data should be found directly from Authors.
